# Data availability in *Chemical Science*

**DOI:** 10.1039/d1sc90105b

**Published:** 2021-05-25

## Abstract

Introducing Data Availability Statements in *Chemical Science*.
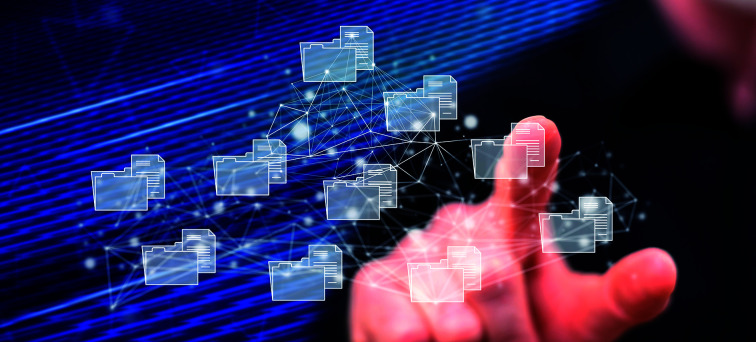

All Royal Society of Chemistry journals, including *Chemical Science,* request that authors make available all the data required to understand and verify the research presented in their article. This is so that the results and conclusions in that article can be accurately assessed by reviewers and editors as part of the peer-review process, and reinforces the robustness and longevity of the conclusions.

Currently we request all authors deposit X-ray crystallographic data in an appropriate repository, such as the Cambridge Structural Database (CSD) or Protein Data Bank (PDB), and suggest authors include all other data within Electronic Supplementary Information (ESI) documents, published as PDFs alongside the final published article.^1^

The current practice to highlight these datasets to readers is to refer to them in the main text of the article, or as a footnote.

In addition to this essential activity, wider data sharing beyond crystal data deposition and use of ESI PDF documents is strongly recommended. *Chemical Science* strongly encourages authors, where possible, to deposit all data associated with the research in a manuscript in external repositories, so that this data is then findable, accessible, interoperable, and reusable (FAIR),^2^ enabling other researchers to replicate and build on that research.

A small number of authors are already including details of where this data can be found in a separate Data Availability section in their manuscript, we want to help make this a more widespread practice.

## What is changing?

In order to encourage high standards of transparency, research reproducibility, and to promote the reuse of new findings, *Chemical Science* is now asking all authors to include a Data Availability Statement as part of the final published article, as standard.

## How is this different from including a footnote?

Data Availability Statements help to formalise the process of data discovery. We are not asking authors to change the way they share data associated with their publications overnight, but aim to improve the visibility of their efforts. We do also hope that the introduction of this measure will further encourage authors to consider wider research data sharing in repositories and data citation, as part of the scientific and publication process.

## What are Data Availability Statements?

Data Availability Statements provide information about where data, software or code supporting the results reported in a published article can be found. It is the section of the paper where readers, funders and even automated algorithms, go to find out where to locate the underlying data, in order to verify claims or re-use for future research.

## Why do we need them?

Data Availability Statements are important because they support the validation of data to maintain high standards of research reproducibility. They increase transparency and encourage trust in the scientific process. They encourage the reuse of new findings, and also normalise the formal citation of data, so thereby ensuring all researchers involved in producing the data can gain credit for their research outputs. They also save researchers time in finding key information from scientific articles.

From a practical perspective, many funding bodies also require the inclusion of Data Availability Statements, so this will support our authors in being compliant with data policies. We are currently developing more detailed advice on which repositories will be suitable for archiving different types of data across the chemical sciences, and this will be available shortly as part of the RSC author guidelines. We hope this will assist our authors in navigating the sometimes complex area of research data management.

## What should I include?

The Data Availability Statement should include, where applicable, links to datasets shared in an external data repository, which have been analysed or generated during the study. This section should list the database, deposition number, DOI, URL or any other relevant details. Authors are also encouraged to formally cite their associated datasets in the reference section of an article.

The Data Availability Statement can also provide information about the data presented in an article itself (*e.g.* in figures or tables), or provide a reason if data is not available to access. If supporting data or code have been included in the article’s ESI, this should also be stated here.

A Data Availability Section should be included at the end of the article, after the Conclusions section.

## Examples of Data Availability Statements

• Crystallographic data for [compound number] has been deposited at the [name of repository, such as CCDC/ICSD/PBD] under [deposition number] and can be obtained from [URL of data record].

• The datasets supporting this article have been uploaded as part of the supplementary information.

• The code for [description of ] can be found at [URL] with [DOI – if available].

• Data for this paper, including [description of data types] are available at [name of repository] at [URL – format https://doi.org/DOI].

If the data are not available, for ethical or commercial reasons, the following examples could be used:

(1) The datasets generated during and/or analysed during the current study are not publicly available due to [insert reason] but are available from the authors on reasonable request.

(2) Data sharing is not applicable to this article as no datasets were generated or analysed during the current study.

(3) The data that support the findings of this study are available from [third party name] but restrictions apply to the availability of these data, which were used under license for the current study, and so are not publicly available. Data are however available from the authors upon reasonable request and with permission of [third party name].

## How will the journal help?

We will aim to make the introduction of Data Availability Statements as simple as possible for authors. We have produced detailed guidance for authors including the template statements for authors to choose from based on what we believe are the most common data sharing practices within chemistry. We are also introducing a reminder for authors, at the revision and proofing stages of the publication process.

Finally, we also provide suggestions for general repositories, and will be following up soon with more detailed subject-specific repositories for data types specific to the chemical sciences. We hope this will help to advise authors on where they can deposit their datasets.^3^

## Acknowledgements and top tips

We should acknowledge the work that many other publishers and journals have already put into developing best practice guidelines around research data, which we have drawn heavily on for inspiration and guidance.^4^

There is also an extremely rich source of advice and top tips available for authors from other publishers, and for some general good advice, we recommend this article for Data Availability Statement top tips!^5^

As ever, we would like to hear from you about how this change impacts you as an author, and how we can help improve this process. Do get in touch at chemicalscience-rsc@rsc.org.

May Copsey,

Executive Editor, *Chemical Science*

## Supplementary Material
